# 

**Published:** 1843-06-24

**Authors:** 


### CONTENTS.

Case of fracture of the scull,—compression of the brain—laceration of the dura mater—escape of cerebral substanoe—depression of the bone—treatment—recovery—and remarks. By John S. Rohrer, M. D. - - - - - 133

*Jefferson Medical College.*

Clinic of Professor Mütter, continued. Polypus of the nose, - - - 135

A list of cases discharged from the surgical wards of the Pennsylvania Hospital, during the month of May, 1843. By Ellerslie Wallace, M. D. - 138

*Bibliographical Notices.*

A demonstration of the curability of pulmonary consumption in all its stages. Comprising an inquiry into the nature, causes, symptoms, treatment, and presentation of tuberculous diseases in general. By Wm. A. McDowell, M. D. Louisville, Ky., 1843, - 139

The kidneys and urine. By J. J. Berzelius. Translated from the German, by M. H. Boyé and F. Leaming, M. D. 140

EDITORIAL—MEDICAL SERVICE IN THE NAVY, - - - - - - - 140

Death of Mr. Tyrrell, - - - 141

*Retrospect of the Medical Sciences.*

Fever with pericarditis, - - - 142

Vesico-uaginal fistula, - - - 142

Fibrous polypus of the uterus, - 144

On electro-puncture in the treatment of deafness, depending on a paralysis of the acoustic nerve. By M. Jobert, - 144

